# miR-221/222-3p act as potential circulating factors in heart failure to stimulate cancer progression

**DOI:** 10.3389/fonc.2025.1615422

**Published:** 2026-01-12

**Authors:** Rongfeng Xu, Jiaqi Guo, Zhenjun Ji, Kongbo Zhu, Zulong Sheng, Pengfei Zuo, Abdlay Carvalho, Yuyu Yao, Genshan Ma, Rui Zhang

**Affiliations:** 1 Department of Cardiology, Zhongda Hospital, School of Medicine, Southeast University, Nanjing, Jiangsu, China; 2 Clinical Research Center for Cardiovascular Diseases, Fuwai Central China Cardiovascular Hospital, Henan Provincial People’s Hospital Heart Center, Zhengzhou University Central China Fuwai Hospital, Zhengzhou, Henan, China

**Keywords:** bioinformatics, cancer, circulating factors, heart failure, miR-221/222-3p

## Abstract

**Background:**

Recent epidemiological studies have shown that heart failure (HF) can lead to an increased incidence of cancer. However, there is limited information regarding how HF promotes cancer development. We attempted to clarify the potential role of miR-221/222-3p dysregulation in HF in promoting cancer through comprehensive application of various bioinformatics tools and *in vitro* validation.

**Methods:**

Various bioinformatics tools, including TargetScanHuman, the Human microRNA (miRNA) tissue atlas, RNA structure, miRNET, DAVID, Enrichr, FunRich, STRING, MalaCards, miRcancer, OncoLnc, miRTargetLink, GEPIA, the cBioportal, the GEO database, and Cytoscape, were comprehensively applied. Twenty patients with coronary artery disease (CAD) admitted to Zhongda Hospital, Southeast University, were enrolled in this study. The patients were divided into HF and non-HF groups. Serum (5% in complete medium) from patients with or without HF was added to the HT-29 cell culture medium. Inhibitors of miR-221/222-3p were constructed. EdU kits and CCK8 kits were used for proliferation detection. Western blotting was used to measure the level of PCNA. qRT-PCR was used to measure the levels of miR-221/222-3p.

**Results:**

miR-221/222-3p was widely distributed in various tissues and organs, including the heart. The PPI network revealed that miR-221/222-3p was closely associated with HF, whereas the KEGG pathway analysis indicated that the functions of miR-221/222-3p were mainly cancer-related. The Cytoscape analysis indicated that miR-221/222-3p act as key regulators of the progression of several common malignant tumors through their target mRNAs. *In vitro* experiments showed that miR-221/222-3p was elevated in the serum of HF patients and in human colon cancer cells (HT-29) treated with HF serum. HF serum promoted the proliferation of HT-29 cells, which was reversed by miR-221/222-3p inhibitors.

**Conclusion:**

miR-221/222-3p may be an important link between HF and cancer, as they are upregulated in HF and thus promote cancer.

## Introduction

In recent past decades, heart failure (HF) and cancer have become the main causes of death worldwide (1). The risk of HF and cancer has consistently increased with increasing age (2). HF and cancer result from prolonged exposure to pathogenic factors. It has been recognized that these individuals share several common risk factors such as obesity, diabetes mellitus, and a sedentary lifestyle (3, 4). Previous studies have also shown that with the development of cancer therapy, the survival time of patients with cancer has gradually increased. This group of patients usually suffer from cardiovascular diseases, which are caused mainly by HF due to the cardiotoxicity of antineoplastic drugs in the later stages (5).

Recent studies have suggested that HF is an important cause of the development and progression of malignancy, and increasing numbers of patients with HF are diagnosed during the follow-up of (6, 7). Meijers et al. (8) clarified that HF stimulates tumor growth mainly through the release of circulating factors from the heart, revealing a mutual concomitant relationship and deepening the understanding of the connection between HF and cancer. The specific mechanism linking these two diseases requires further investigation.

MicroRNAs (miRNAs), a type of noncoding RNA molecule 22 nucleotides (nt) in length, can negatively regulate the expression of target mRNAs by inhibiting translation or promoting mRNA degradation (9). miRNAs have been proven to participate in a variety of biological functions, including apoptosis, proliferation, migration, and inflammation (10). miRNA-221-3p and miRNA-222-3p are two highly homologous miRNAs that are significantly dysregulated in several human diseases (11), including HF (12) and cancer (13). miR-221-3p/222-3p are selected for this study because of their high homology, abnormal upregulation in both HF and cancer, clear functional roles, and ability to synergistically regulate disease-related pathways—factors that make them ideal candidates to explore the link between HF and cancer.

Based on bioinformatics analysis and in vitro experimental validation, we aimed to better understand the roles of miR-221/222-3p and their regulatory network in linking HF and cancer.

## Materials and methods

### Analysis of miRNA sequences, secondary structures, and tissue distribution profiles

The TargetScanHuman 7.2 database (http://www.targetscan.org/vert_72/) (14) was used to predict the same seed sequence and consequential pairs of target regions of miR-221/222-3p. The miRbase database (http://www.mirbase.org/) (15) was used to acquire stem-loop sequences, and the RNA structure (http://rna.urmc.rochester.edu/RNAstructureWeb/) was used to construct stem-loop structures. The human miRNA tissue atlas (https://ccb-web.cs.uni-saarland.de/tissueatlas/) (16) was used to determine the expression of miR-221/222-3p in normal tissues.

### Analysis of validated and predicted target mRNAs of miR-221/222-3p

As an integrated platform linking miRNAs and target genes, the miRNET database (https://www.mirnet.ca/) (17) integrates data from the following miRNA databases: miRTarBase, miRecords, miRanda, miR2Disease, HMDD, PhenomiR, SM2miR, DIANA-TarBase, PharmacomiR, EpimiR, and StarBase but not from TargetScan. Therefore, we combined miRNET and TargetScanHuman to search for the downstream target genes of miR-221/222-3p. In addition, the intersecting genes of both miR-221-3p and miR-222-3p screened by miRNET were further analysed via the DAVID 6.8 database (https://david.ncifcrf.gov/) to determine tissue locations (18).

### Analysis of the biological role of the target genes of miR-221/222-3p

The Kyoto Encyclopedia of Genes and Genomes (KEGG, https://www.kegg.jp/) is a database used to understand the high-level functions and utilities of biological systems. The enrichment database (https://amp.pharm.mssm.edu/Enrichr/) (19) was used to perform the KEGG analysis. The Catalog of Somatic Mutations in Cancer (COSMIC, https://cancer.sanger.ac.uk/cosmic) is a comprehensive database that contains information on somatic mutations in multiple human cancer types. Funrich software (http://www.funrich.org/) (20) is a functional enrichment analysis tool used to perform ‘COSMIC’ analysis.

### Construction and analysis of the protein-protein interaction network

STRING (https://string-db.org/) (21), the most widely used database for exploring proteins and their functional interactions, was used to construct a complete PPI network. Cytoscape (https://cytoscape.org/) (22) is an open-source software platform for visualizing complex networks and hub genes among networks selected using the ‘CytoHubba’ plug-in.

### Potential role of miR-221/222-3p in human cancers

A literature search for potential regulatory oles of miR-221-3p and miR-222-3p in cancers was based on PubMed (https://www.ncbi.nlm.nih.gov/pubmed) and the miRCancer database (http://mircancer.ecu.edu/) (23). The Cancer Genome Atlas (TCGA, https://portal.gdc.cancer.gov/) is the most extensive cancer genomics programme that describes the molecular characteristics of more than 20,000 primary cancers and matched normal samples spanning 33 cancer types. OncoLnc (http://www.oncolnc.org/) is a web tool that provides easy access to TCGA data and is widely used to analyse the survival rate of patients with different cancers stratified by miRNA expression. The MiRTargetLink database (https://ccb-compute.cs.uni-saarland.de/mirtargetlink2) provides miRNAs with target genes verified by experiments (24). Gene Expression Profiling Interactive Analysis (GEPIA, http://gepia.cancer‐pku.cn/) is another database used to analyse the survival rate of mRNAs in TCGA data (25). cBioportal (http://www.cbioportal.org/) was used to research applications, mutations, and deletions of target genes in the TCGA database (26).

### Confirmation of the role of miR-221/222-3p in regulating HF related cancers

The mRNA array dataset of colorectal cancer (GSE126092) was obtained from the GEO database (https://www.ncbi.nlm.nih.gov/geo/), and mRNA expression levels were analysed using the GEO2R online tool to identify differentially expressed genes (DEGs) (https://www.ncbi.nlm.nih.gov/geo/geo2r/). The screening criteria for DEGs were as follows: fold change (FC) > 1 and adjusted P < 0.05. The PPI network was constructed using STRING and the hub genes were selected out by the cytoHubba plug-in of Cytoscape according to ‘Degree’.

### Patient enrollment

The clinical study in this project was approved by the IEC for Clinical Research of Zhongda Hospital, affiliated with Southeast University (2021ZDSYLL167-P01). Research involving human research participants was performed in accordance with the Declaration of Helsinki. Twenty patients with coronary artery disease (CAD) from Zhongda Hospital, Southeast University were enrolled in this study. The inclusion criteria for patients were as follows: (1) aged older than 18 years and younger than 75 years and (2) diagnosed with HF-reduced ejection fraction (HFrEF) because of ischaemic heart disease. The exclusion criteria for patients were as follows: (1) had severe hepatic and kidney dysfunction; (2) had autoimmune diseases, tumor diseases or severe pulmonary diseases; (3) were pregnant; and (4) were deemed inappropriate by researchers. The patients were divided into HF and non-HF groups (**Supplementary Table 1**). Written informed consent for participation in the study was obtained.

### Cell culture and transfection

HT-29 cells (human colon cancer cells) were purchased from Procell Company (CL-0118, Wuhan, China). Serum from HF and non-HF groups was pooled equally due to limited individual serum volume, serum (5% in complete medium) from patients with or without HF was added to the HT-29 cell culture for 2 h (at 37 °C and 5% CO2). miR-221-3p and miR-222-3p inhibitors (Antisense oligonucleotides, ASOs) were transfected into cells with RNATransMate (Sangon Biotech, Shanghai, E607402). The experiment set non-inhibitor HF serum-treated group as control to verify the specific role of miRNAs. When the cell confluence reached 60-90%, RNATransMate and RNA were added to tubes A and B, which contained serum-free culture medium and diluted. Then, tubes A and B were mixed together. The RNA/RNATransMate complex mixture was allowed to stand at room temperature for 5–10 minutes. After that, the RNA/RNATransMate mixture was added to the wells.

### Cell proliferation

A CCK-8 (CK04, Dojindo, Japan) and BeyoClick™ EdU Cell Proliferation Kit with Alexa Fluor 594 (C0078S, Beyotime, China) were used to detect cell proliferation. The 96-well plate culture plates were preincubated with serum in an incubator for 48 h (at 37 °C and 5% CO2). 10μl CCK-8 solution was added to each well. The plates were incubated for 1–4 hours. The absorbance was measured at 450 nm using a microplate reader. For EdU staining (C0078S, Beyotime, China), EdU working solution preheated at 37 °C was added to the confocal dish (10 μM) and incubated for 2 h. The culture medium was then removed, and 4% paraformaldehyde was added for 15 min at room temperature. After fixation and washing, the penetrating solution was added at room temperature for 10 min. Then, the Click reaction solution was added to each well and the samples were incubated in the dark at room temperature for 30 min. Finally, the nuclei were stained using a Hoechst kit. A confocal microscope (A1RHD25, Nikon, Japan) was used to observe the fluorescence.

### Real-time quantitative PCR

RNA was extracted using the TRIzol reagent (15596018, Invitrogen, USA). A miRNA 1st Strand cDNA Synthesis Kit (by stem-loop) (MR101, Vazyme, China) was used for cDNA synthesis. A ChamQ Universal SYBR qPCR Master Mix Kit (Q711; Vazyme, China) was used for qRT-PCR. All procedures were performed according to the manufacturer’s instructions. The sequences of hsa-miR-221-3p were as follows: RT-primer, GTCGTATCCAGTGCAGGGTCCGAGGTATTCGCACTGGATACGACGAAACC; forward primer, AACGGCAGCTACATTGTCTGCT; reverse primer ATCCAGTGCAGGGTCCGAGG. The sequences of hsa-miR-222-3p were as follows: RT-primer, GTCGTATCCAGTGCAGGGTCCGAGGTATTCGCACTGGATACGACACCCAG; forward primer, AGCGCCTAGCTACATCTGGCT; and reverse primer, ATCCAGTGCAGGGTCCGAGG.

### Western blotting

Total cell protein was extracted with a protein extraction kit (KGP2100, KeyGEN BioTECH). The protein concentration was detected using a BCA Protein Assay Kit (KGP902, KeyGEN BioTECH). Proteins were separated using SDS-PAGE and the target proteins were transferred to the PVDF membranes. The membrane was blocked with 5% skimmed milk at 4°C for 90 minutes. The membrane was washed with TBST 3 times for 5 minutes each and then incubated with primary antibodies overnight at 4°C in a shaker. Next, the membrane was washed 3 times for 5 minutes each, and incubated with the corresponding secondary antibodies at room temperature for 1 hour. After being washed with TBST 3 times for another 5 minutes each, the membrane was exposed by an automated chemiluminescence imaging analysis system (Tanon 5200, Shanghai).

### Statistics

Data were analyzed using SPSS 25.0 (USA) and GraphPad Prism 8 (USA). Continuous variables are presented as mean ± SD. Comparisons between two groups were performed using independent samples t-tests or rank-sum tests. Categorical variables were compared using χ² tests or Fisher’s exact tests. ANOVA was used for inhibitor intervention experiments. Multiple comparisons were corrected using the Benjamini-Hochberg method (FDR < 0.05). Effect sizes (Cohen’s d, OR) and 95% confidence intervals (CI) were reported for key outcomes.

## Results

### The characteristics of hsa-miR-221/222-3p

PubMed miRNA search revealed that “miR‐221” and “miR‐222” are the most commonly used terms names in a variety of studies. The term miR-221/222 serves as the family designation, and their precursors can be processed into two mature isoforms: 3p and 5p. Among these, the 3p isoforms are more frequently documented in the literature and were more prominent in our preliminary analysis, with their functions being better characterized. Therefore, the subsequent experiments will focus specifically on miR-221-3p and miR-222-3p. Similarly, both miR-221-3p and miR-222-3p have mature sequences and are suitable for follow-up research. The mature sequences of miR-221-3p and miR-222-3p in humans are shown in **Supplementary Figure 1A**, and one target gene, CDKN1B, was used as an example of all the common target genes of both miR-221 and 222-3p. In addition, considering that the stem‐loop structure of precursors is critical for processing into miRNA‐3p or 5p, we further searched for pre-miR-221/222 sequences and subsequently constructed an RNA secondary structure by the ‘RNA structure’ presented in **Supplementary Figure 1B**. The expression levels and distribution ranges of miRNAs in different tissues and/or organs usually indicate their different roles in tissue development and disease. Next, we analyzed the tissue location of miR-221/222-3p with the ‘human miRNA tissue atlas,’ and the results showed that miR-221/222-3p was widely distributed in the bladder, brain, epididymis, colon, lung, prostate, and heart (**Supplementary Figures 2**, **3**), indicating a potentially wide range of biological functions for miR-221/222-3p. We confirmed miR-221-3p/222-3p as the core research objects, and all subsequent experiments focused on their expression and function.

### Target genes of miR-221/222-3p predicted by miR-NET

**Figure 1:**
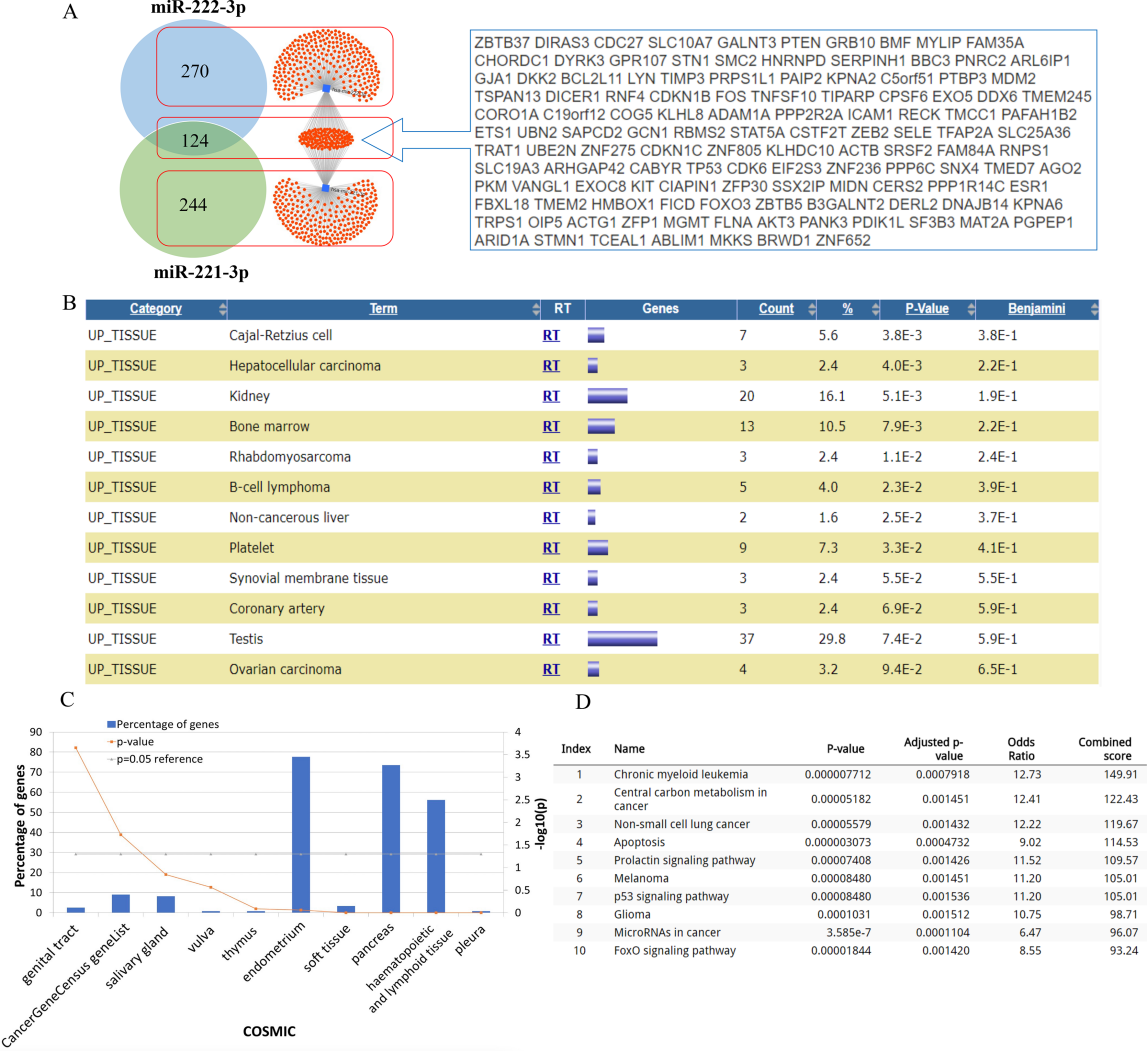
Target genes of miR-221-3p and miR-222-3p and their location in tissues. **(A)** Predicted genes by miRNET showed that 124 genes (in cyan frame) were targeted by both miR-221-3p and miR-222-3p while 244 and 270 genes by miR-221-3p and miR-222-3p, respectively. **(B)** Top 10 tissue distribution of common 124 target genes showed by DAVID. **(C)** Top 10 results of COSMIC analysis by FunRich. **(D)** KEGG analysis presented the top 10 enriched pathways.

It is well known that miRNAs usually target mRNAs to inhibit their expression, and thus exert multiple biological functions. Therefore, identifying potential target genes of miRNAs is important. The miRNET webtool includes 11 miRNA-related databases and provides reliable results. We found that 368 genes were targeted by miR-221-3p while 394 were targeted by miR-222-3p. Intersecting these two groups of genes revealed that almost one-third of the genes (124) were targeted by the two miRs (**Figure 1A**). The biological function of miRNAs is mediated by the mRNAs of target genes. As shown in **Figure 1B**, these 124 cotargeted genes were widely distributed in various tissues, including the kidney, bone marrow, and testis, indicating that miR-221/222-3p has multiple regulatory functions in the human body.

To reveal the specific biological function of miR-221/222-3p, we performed KEGG analysis of 124 target genes to comprehensively evaluate the role of these miRNAs. These results suggest that miR-221/222-3p and its downstream genes are closely related to cancer. The COSMIC database is a comprehensive database of mutations in cancer genes. Therefore, we further analysed the 124 targeted genes in the COSMIC database, and the results showed that genes were only enriched in the genital tract and were enriched in the cancer gene (**Figure 1C**). The results showed that miR-221/222-3p was significantly correlated with chronic myeloid leukemia, central carbon metabolism in cancer, non-small cell lung cancer, apoptosis, and the prolactin signalling pathway (**Figure 1D**).

### Target genes of miR-221/222-3p predicted by TargetScan

**Figure 2:**
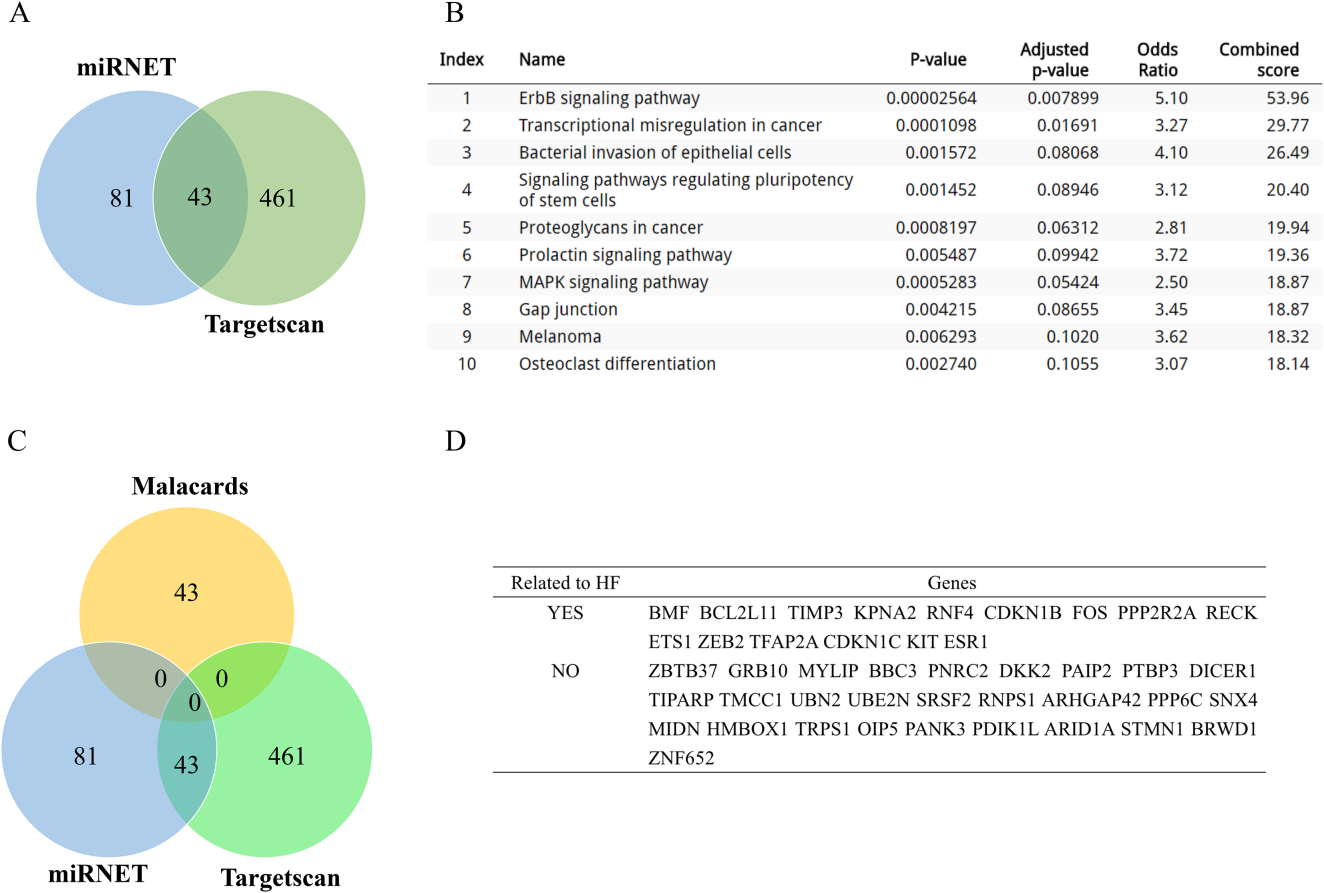
Function analysis of targeted genes provided by another tool Targetscan and comprehensive analysis of heart failure-related genes provided by Malacards database. **(A)** Venn diagram showed 43 genes were predicted to be targeted by miR-221/222-3p both in miRNET and Targetscan, and 461 genes were only predicted in Targetscan. **(B)** KEGG analysis presented the top 10 enriched pathways of 461 genes. **(C)** Venn diagram showed common genes of miRNET, Targetscan and Malacards, and the 43 heart failure-related genes of Malacards cannot be targeted by miR-221/222-3p directly. **(D)** Statistics of heart failure related genes by literature search of 43 intersected genes between miRNET and Targetscan indicated 15 genes were potentially heart failure related.

TargetScan is a web program that provides prediction results for miRNA target genes in humans. Considering that miRNET excludes the potential results of TargetScan, we analyzed miRNET and TargetScan. First, we predicted the genes likely to be targeted by both miR-221 and 222-3p and identified 504 potential genes. Second, we screened the intersection with the miRNET results, and 43 genes were selected by both miRNET and TargetScan, while 461 genes were only predicted by only TargetScan (**Figure 2A**). Moreover, we constructed a PPI network of 43 intersecting genes, and the results revealed a close relationship among these genes (**Supplementary Figure 4**). Finally, we performed KEGG analysis of the remaining 461 genes to fully understand the function of miR-221/222-3p. The ErbB signalling pathway and transcriptional dysregulation in cancer were important components of the KEGG pathway (**Figure 2B**). These results confirmed that miR-221/222-3p is closely related to cancer.

### Emerging role of miR-221/222-3p in HF

Several pathological processes, such as cardiac fibrosis and hypertrophy related to HF, are regulated by a series of miRNAs (27), especially miR-221-3p and miR-222-3p (12, 28). Malacards is an integrated database that includes genes related to various diseases. To further evaluate the role of miR-221/222-3p in HF, we screened the database with the keyword “HF” and obtained a total of 43 genes from the Congestive HF, Systolic HF, and Diastolic HF clauses. Next, we intersected these 43 genes with miR-221/222-3p target genes that were predicted by miRNET and TargetScan. However, the results (**Figure 2C**) showed that no genes coexisted in the three groups or even in the malacards according to the miRNET or Targetscan results. Therefore, we searched for 43 genes targeted by miRNET and TargetScan in PubMed to identify potential HF-related genes. Fifteen genes, including BMF, BCL2L11, and TIMP3, were found to have potential functions in HF (**Figure 2D**). We also constructed a PPI network of a combination of 15 (PubMed searched) and 43 (Malacards searched) HF genes. The complex network structure (**Supplementary Figure 5**) indicated strong interactions between these 58 genes and showed that miR-221/222-3p could directly or indirectly regulate HF development.

### miR-221/222-3p and their target genes in human cancers

**Figure 3:**
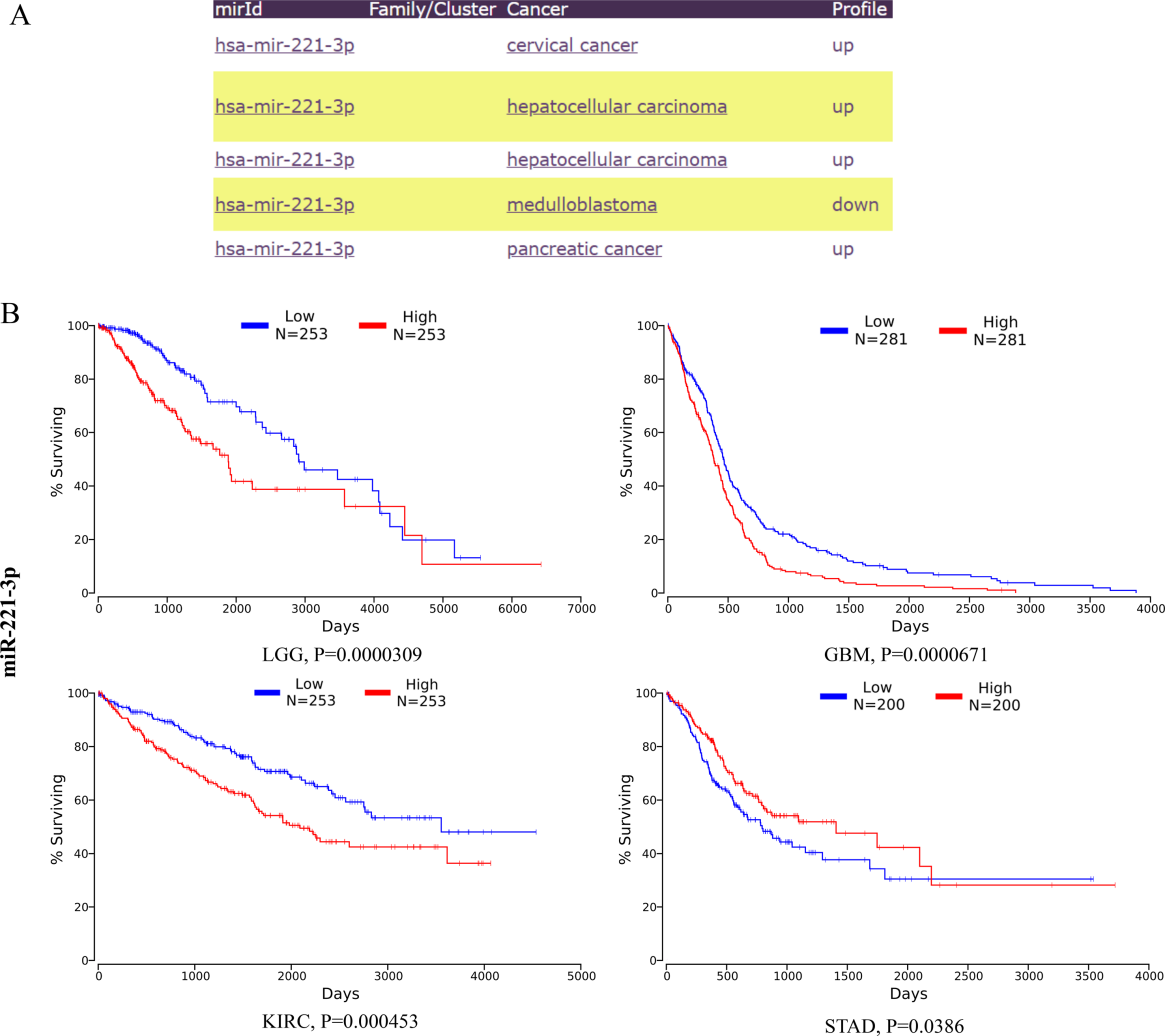
Role of miR-221-3p in human cancers. **(A)** miRCancer provides expression patterns of miR-221-3p in several cancers. **(B)** OncoLnc survival analysis of miR022103p in diûerent human cancers. LGG, low-grade glioma; GBM, Glioblastoma multiforme; KIRC, kidney renal clear cell carcinoma; STAD, Stomach adenocarcinoma; KIRP, Kidney renal papillary cell carcinoma; CESC, Cervical squamous cell carcinoma and endocervical adenocarcinoma; BRCA, Breast invasive carcinoma.

**Figure 4:**
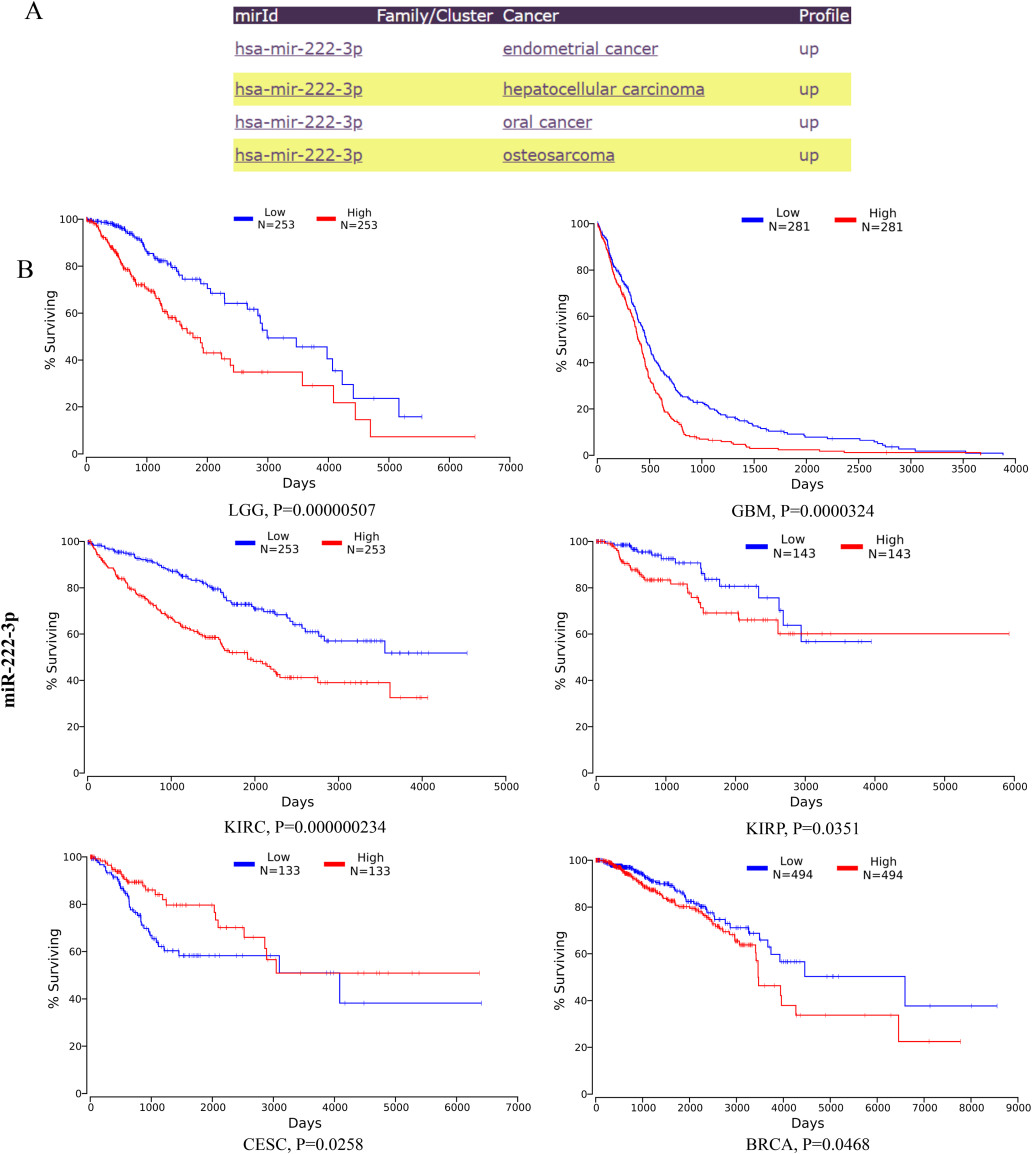
Role of miR-222-3p in human cancers. **(A)** miRCancer provides expression patterns of miR-222-3p in several cancers. **(B)** OncoLnc survival analysis of miR-222-3p in diûerent human cancers. LGG, low-grade glioma; GBM, Glioblastoma multiforme; KIRC, kidney renal clear cell carcinoma; STAD, Stomach adenocarcinoma; KIRP, Kidney renal papillary cell carcinoma; CESC, Cervical squamous cell carcinoma and endocervical adenocarcinoma; BRCA, Breast invasive carcinoma.

KEGG analysis of the target genes identified by miRNET and TargetScan revealed that these genes were significantly enriched in cancer-related pathways, indicating the critical role of miR-221/222-3p in cancer. To confirm the connection between miR-221/222-3p and cancer, we performed a literature search in PubMed and identified 62 articles related to ‘miR-221-3p and cancer’ and 126 articles related to ‘miR-221-3p’, while 62 and 110 articles related to ‘miR-222-3p and cancer’ and ‘miR-222-3p’, respectively, suggested that miR-221/222-3p is an important tumor factor. The miRcancer database is a literature-supported miRNA-cancer database. The expression of miR-221/222-3p was dysregulated in cervical cancer, hepatocellular cancer, medulloblastoma cancer, and endometrial cancer (**Figures 3A**, **4A**). Therefore, we performed a survival analysis of patients with diverse cancers stratified according to the miR-221/222-3p signature using the OncoLnc database in the TCGA cohort. Kaplan-Meier (KM) curves (**Figures 3B**, **4B**) showed that high levels of miR-221-3p and miR-222-3p usually resulted in poor overall survival (OS) in most cancers, except for STAD and CESC, which indicated the dual effects of miR-221/222-3p in different cancer types.

**Figure 5:**
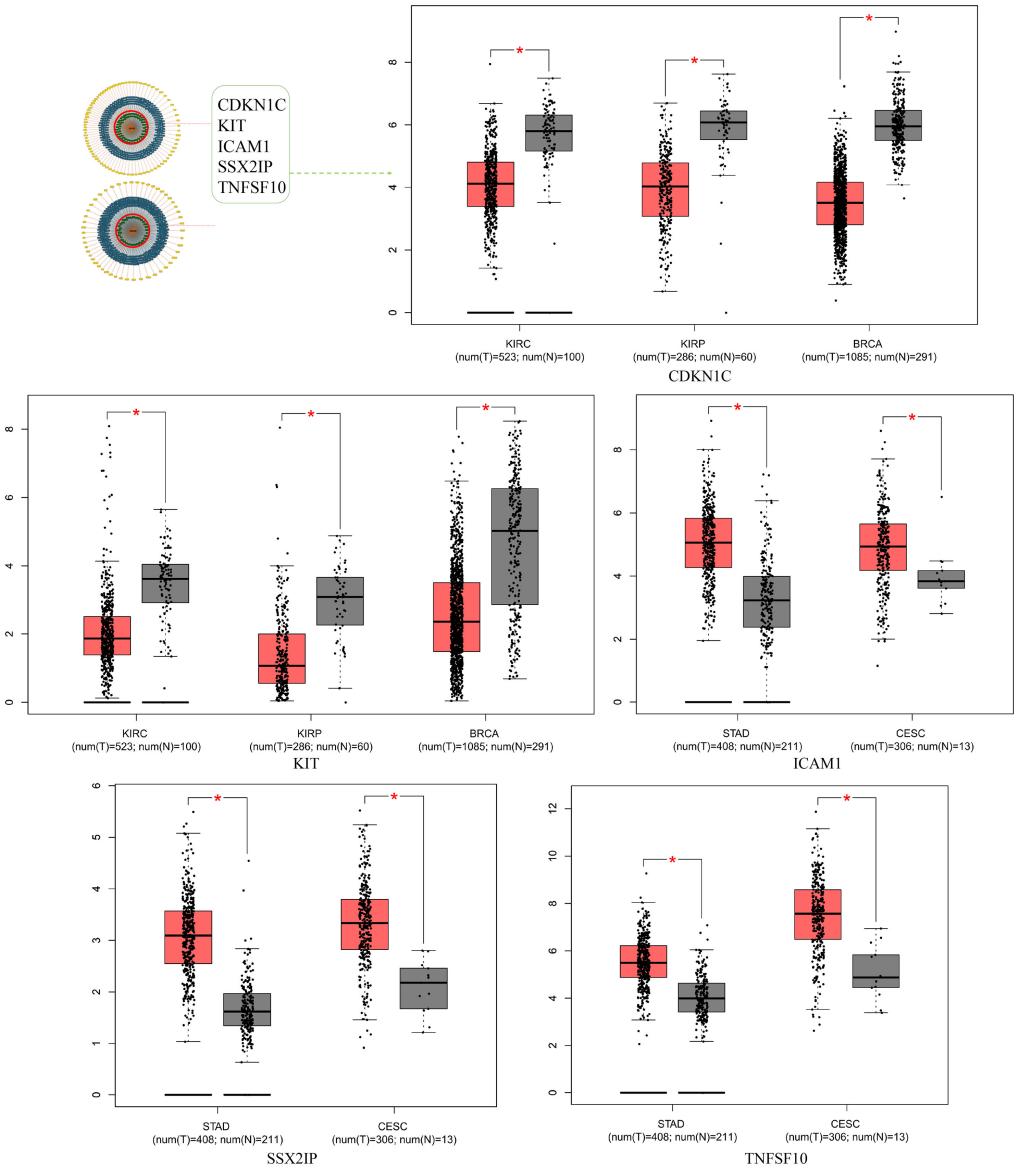
Role of miR-221/222-3p targeted genes in human cancers. Five genes from experimentally validated in strong evidence (in the circle) provided by MiRTargetLink were selected as candidates to detect expression in several cancers.

**Figure 6:**
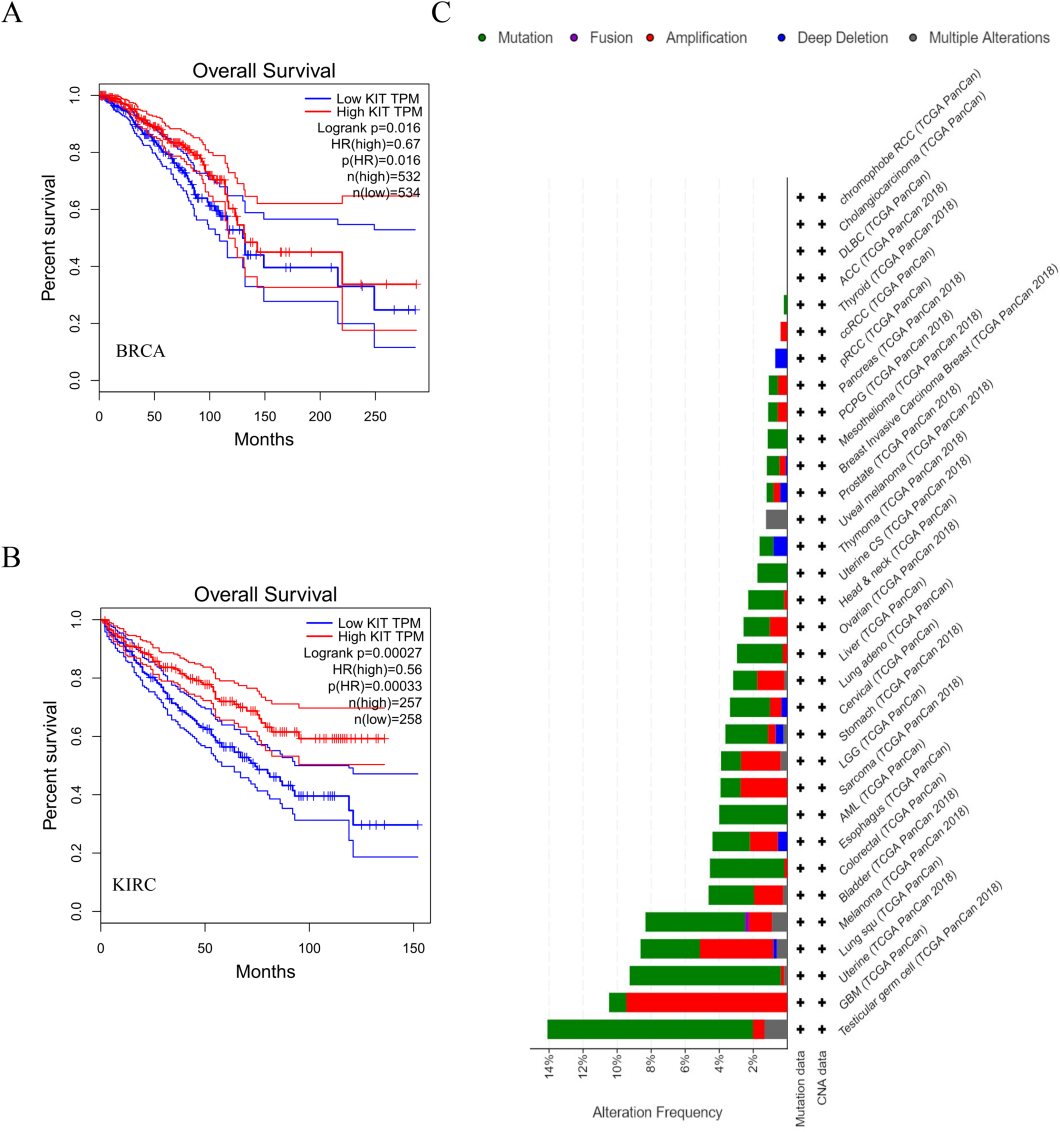
Role of miR-221/222-3p targeted gene KIT in human cancers. **(A, B)** Gene KIT was a candidate to analyze survival in two cancers by GEPIA. **(C)** cBioportal provided information on somatic mutations analysis of KIT gene.

To further understand the specific role of miR-221/222-3p in regulating cancer, the expression of five experimental validated target genes (CDKN1C, KIT, ICAM1, SSX2IP, and TNFSF10) from the miRTargetLink Human database (**Figure 5**) among several previous cancers was calculated via the ‘GEPIA’. Consistently, the expression of CDKN1C and KIT was lower in cancers, including KIRC, KIRP, and BRCA, while ICAM1, SSX2IP, and TNFSF10 were higher in cancers, including STAD and CESC, opposing the effects of miR-221/222-3p (**Figure 5**). KIT was selected as an example for survival analyses for patients in BRCA and KIRC. The results showed that low expression of KIT had an adverse effect on the survival of cancer patients, which was contrary to the effects of miR-221/222-3p in BRCA and KIRC (**Figures 6A, B**). Finally, somatic mutations in KIT itself are the main cause of cancer (**Figure 6C**), but this finding suggests that the function of KIT in different types of cancer is partly regulated by miR-221/222-3p.

### miR-221/222-3p are key regulators of colorectal cancer

Patients with HF have been demonstrated to have a higher risk of cancer. Neurohormonal activation or proteins secreted from the failing heart are important mechanisms linking these two diseases (29). In addition, a failing heart usually generates a large amount of abnormal noncoding RNAs, including long noncoding RNAs (lncRNAs) and microRNAs (miRNAs), compared to normal hearts (30). Previous studies have shown that miR-221 and miR-222 are highly expressed in the peripheral blood of patients with ischaemic HF (IHF) (31, 32). Meijers et al. (8) found that post-infarction HF could significantly stimulate intestinal tumor growth *in vivo*. Theoretically, high levels of miR-221/222-3p could mediate the tumor-promoting effects of HF, especially ischaemia.

**Figure 7:**
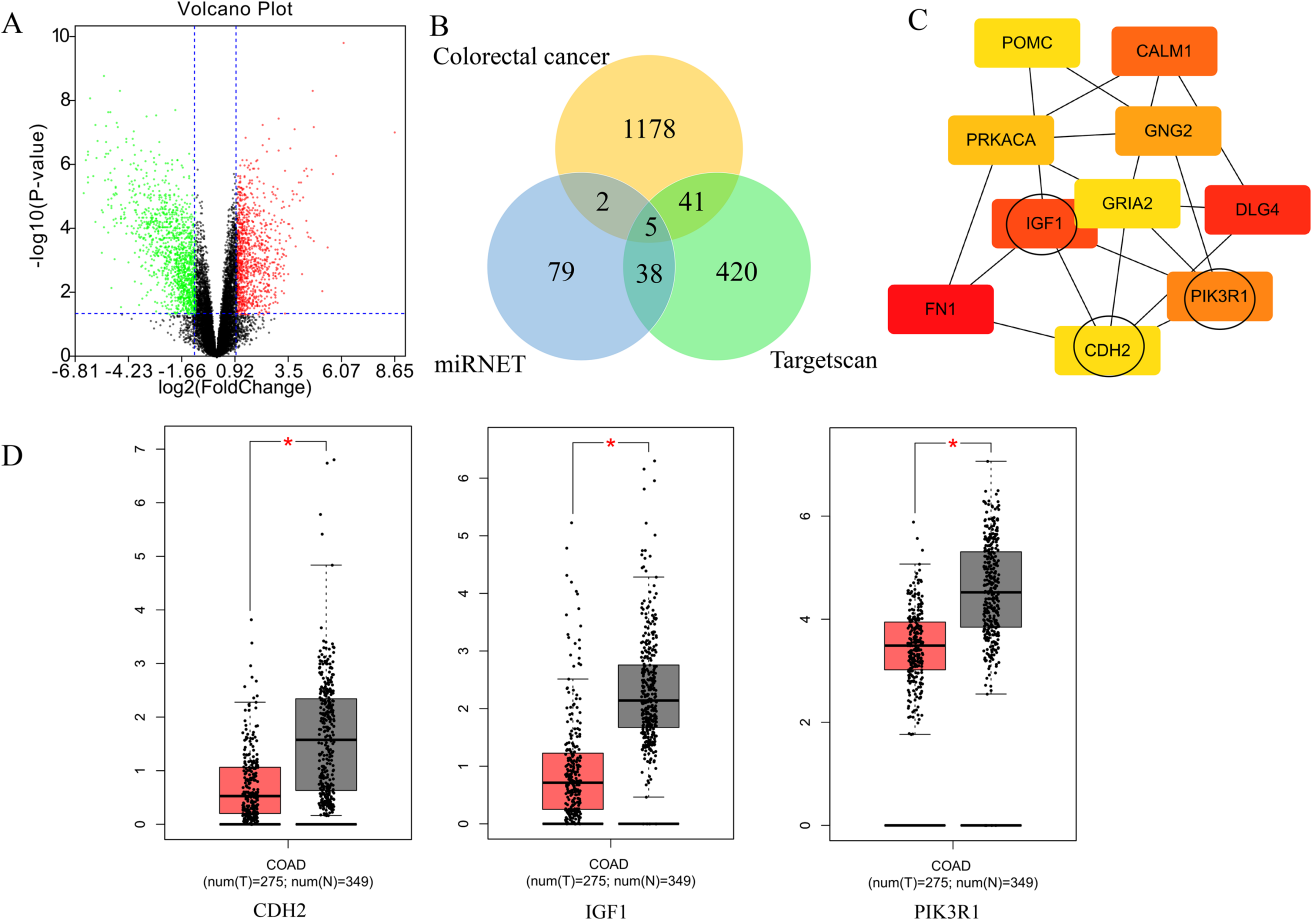
The crucial role of miR-221/222-3p in colorectal cancer. **(A)** The volcano map of GSE126092 indicated signiûcantly down-regulated and up-regulated genes in colorectal cancer. **(B)** Venn diagram showed the intersection among GSE126092 downregulated genes, miRNET and Targetscan targeted genes. **(C)** CytoHubba plug-in of cytoscape ûltered top 10 key genes from the whole PPI network according to the degree score (red: high score; yellow: low score), and three genes (inside the black circle) were predicted to be targeted by miR-221/222-3p directly. **(D)** Three miR-221/222-3p targeted hub genes expression in COAD of TGCA. *p<0.05.

Therefore, we selected colorectal cancer as a candidate disease to assess the core role of miR-221/222-3p in cancer. The GSE126092 dataset (10 human colorectal cancer tissues and 10 corresponding normal-appearing tissues) was screened from the GEO database and analysed using GEO2R. The dataset included lncRNA and mRNA data simultaneously and 1226 downregulated genes after deleting unqualified data (**Figures 7A, B**). Intersection with target genes provided by miRNET and TargetScan revealed that 48 downregulated genes (accounting for approximately 4% of the 1226 genes) were directly regulated by miR-221/222-3p, suggesting that these genes play an important role in colorectal cancer. The PPI network of all downregulated genes revealed an intricate network in which most proteins could interact with a variety of other proteins. In contrast, only a few proteins did not participate in this network (**Supplementary Figure 6**), indicating both direct and indirect regulation of miR-221/222-3p in colorectal cancer. To illustrate the vital role of miR-221/222-3p in colorectal cancer, we used the cytoHubba plug-in of Cytoscape software to perform a deep analysis of the PPI network and selected the top 10 genes according to degree score from high to low (**Figure 7C**). Intriguingly, 3 of the genes (IGF1, PIK3R1, and CDH2) of the 10 were targets of miR-221/222-3p, indicating that miR-221-3p and miR-222-3p play undeniable roles in colorectal cancer. Moreover, the expression of these three genes was all significantly lower in colon adenocarcinoma (COAD) patients in the TCGA database, consistent with the trend obeserved in the GSE dataset (**Figure 7D**). The above bioinformatics results further verify that miR-221/222-3p is involved in an active regulatory network in colorectal cancer, thus providing the justification for our use of HT-29 cells to investigate the ‘heart failure-induced cancer’ mechanism.

### miR-221/222-3p was elevated in the serum of patients with HF and in human colon cancer cells treated with serum from patients with HF

**Figure 8:**
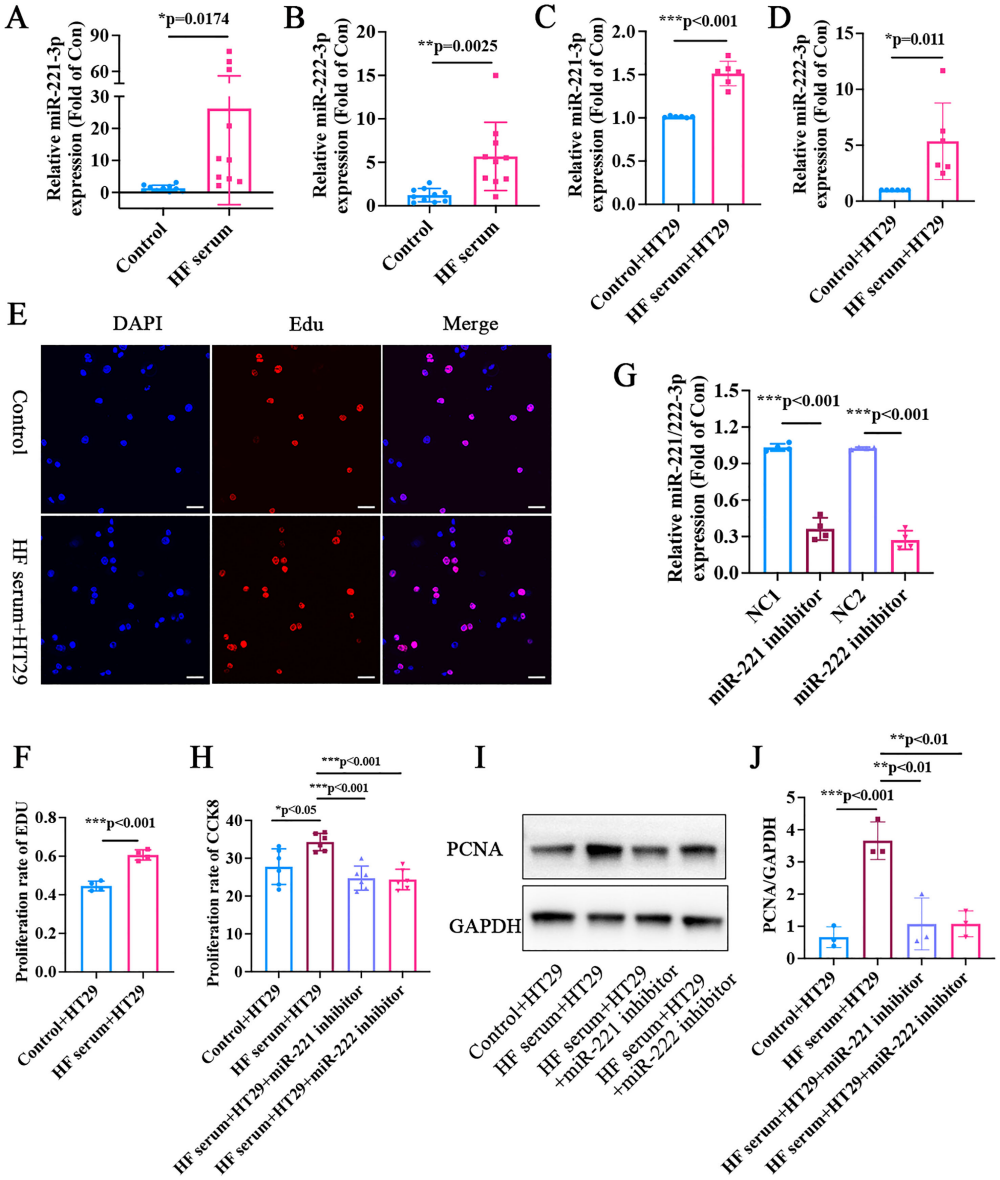
*In vitro* experiments validated the roles of miR-221/222-3p between heart failure and human colon cancer. **(A)** qRT-PCR showed that miR-221-3p was elevated in the serum of patients with heart failure. **(B)** qRT-PCR showed that miR-222-3p was elevated in the serum of patients with heart failure. **(C)** qRT-PCR showed that, compared with HT-29 cells treated with normal serum, miR-221-3p was elevated in cells treated with serum from heart failure patients. **(D)** qRT-PCR showed that, compared with HT-29 cells treated with normal serum, miR-222-3p was elevated in cells treated with serum from heart failure patients. **(E, F)** showed that compared with HT-29 cells treated with normal serum, Edu staining showed a signiûcantly higher proliferation rate in cells incubated with heart failure serum. Red: positive cells, Blue: cell nuclei. Scale bar = 20μm, Count positive cells using ImageJ. **(G)** qRT-PCR showed that, transfection with miR-221-3p and miR-222-3p inhibitors significantly reduced the intracellular levels of miR-221-3p and miR-222-3p, respectively, confirming the efficacy of the knockdown. **(H)** CCK8 measurement showed that serum from patients with heart failure promoted the proliferation of human colon cancer cells, which were reversed by miR-221-3p and miR-222-3p inhibitors, Y-axis: relative proliferation rate (% of control), representing the relative absorbance change in the CCK-8 assay. **(I, J)** Western blot showed that PCNA expression was increased in cells treated with serum from heart failure patients, which was reversed by miR-221-3p and miR-222-3p inhibitors. *p<0.05, **p<0.01, ***p<0.001.

Twenty patients with CAD were enrolled, and divided into HF group and non-HF group (no tumor patients included, per exclusion criteria). qRT-PCR showed that miR-221/222-3p levels were elevated in the serum of patients with HF (**Figures 8A, B**). Compared to those in HT-29 cells treated with normal serum, miR-221/222-3p levels were elevated in cells treated with HF serum (**Figures 8C, D**).

### Serum from patients with HF promoted the proliferation of human colon cancer cells

HT-29 cells were treated with serum from patients with or without HF. CCK-8 and EdU staining revealed that, compared with those in HT-29 cells treated with normal serum, the proliferation rate was significantly greater in cells incubated with serum from HF patients (**Figures 8E, F, H**). Then, HT-29 cells were transfected with miR-221-3p and miR-222-3p inhibitors (**Figure 8G**). Compared to those in the HT-29 cells treated with HF serum, there were significant decreases in PCNA expression and proliferation in the miR-221-3p- and miR-222-3p-inhibiting groups (**Figures 8H-J**).

## Discussion

This study integrated bioinformatics analysis and in vitro experiments to systematically explore the role of miR-221/222-3p in the association between heart failure (HF) and cancer. Our key findings demonstrate that miR-221/222-3p is significantly upregulated in the serum of HF patients and in human colon cancer HT-29 cells treated with HF serum, and that HF serum promotes HT-29 cell proliferation through miR-221/222-3p. These results collectively support the hypothesis that miR-221/222-3p may serve as a critical circulating mediator linking HF to cancer progression.

miR-221/222 are noncoding, single-stranded RNAs located on chromosome Xp11.3. The miR-221/222 genes are transcribed into primary pri-miR-221/222 through RNA polymerase II and then processed into the stem-loop precursor miR-221/122 through the nuclear RNase Drosha in the nucleus. Pre-miR-221/222 is transported to the cytoplasm through the exportin-5 transporter, which is then cleaved into double-stranded miRNA by the nuclease Dicer. The 5’ end of pre-miR221/222 generates miR-221/222-3p. Arginine (AGO) family members bind mature miRNAs to form RNA-induced silencing complexes (RISCs) (33).

miRNAs play an important role in the development and progression of cancer. miR−221/222−3p has multiple functions in the proliferation, apoptosis, invasion, and migration of cancer cells, as well as in tumor microenvironment regulation. miR-221/222-3p promotes the proliferation and metastasis of hepatocellular carcinoma (HCC), estrogen receptor (ER)-positive breast cancer cell lines (33, 34), glioma (35), pancreatic cancer (36), and colon cancer. High expression of miR-221 enhances the invasion and metastasis of colon cancer cells (CRC) by targeting CDKN1C and RECK. Inhibition of miR−222−3p can promote apoptosis in doxycycline (DOX)-resistant CRC. Among these isoforms, the 3p subtype (miR-221-3p/222-3p) is the functionally dominant form in most diseases, including HF and cancer, which justifies our focus on this subtype (37, 38). Because of the role of miR−221/222−3p in colorectal cancer, we selected the HT-29 tumor cell line as the representative tumor cell line to verify the relationship between HFs and tumors.

In the context of HF, accumulating evidence highlights the pathological role of miR-221/222-3p. miR-221/222 were significantly increased in patients with acute viral myocarditis caused by coxsackievirus B3 (CVB3), and inhibiting miR-221/222 aggravated heart injury and inflammation in mice (39). The expression level of miR-221-3p in AMI patients was significantly increased, negatively correlated with LVEF, and positively correlated with the SYNTAX score (40). miR-221 is elevated in HF mice and promotes HF by modulating the p27/CDK2/mTOR axis (41). miR-221/222 appears to exert combined effects on HF and cancer. In a DOX-induced cardiac toxicity mouse model, miR-221/222 was found to be significantly upregulated (42). In this study, we enrolled 20 patients with HFrEF and found that miR-221/222-3p was obviously elevated in HF patients, therefore, there was no need to further expand the sample size of HF patients. Moreover, we treated HT-29 cells with HF serum, and enhanced proliferation of tumor cells was observed.

A critical unresolved question is the transport mechanism of miR-221/222-3p from HF serum to cancer cells. Circulating miRNAs are primarily protected from degradation by encapsulation in extracellular vesicles (EVs), binding to proteins (e.g., AGO2), or association with lipoproteins (43). Among these, exosomes - small EVs (40–160 nm) secreted by nearly all cell types - are particularly attractive candidates for mediating intercellular miRNA transfer (44). Exosomes derived from cardiac cells have emerged as key mediators of systemic signaling in HF. For example, in patients with AMI, exosomes released by damaged cardiomyocytes carry miR-1 and miR-133a into the circulation, where they regulate distant cell functions (45). Sohn et al. extracted exosomal miRNAs from HCC tissues and reported that miR-221/222 levels were significantly greater in the serum of HCC patients than in that of chronic hepatitis B (CHB) and liver cirrhosis (LC) patients (46). In our study, high expression of miR-221/222-3p in the serum of patients with HF promoted the proliferation of human colon cancer cells, which was validated by miR-221/222-3p inhibitors. miR-221/222-3p may be present in exosomes in the serum of patients with HF.

This study has several limitations. We utilized combined bioinformatics analysis to clarify whether miR-221/222-3p can regulate HF progression and cancer. To elucidate the process by which HF promotes colon cancer, in vitro experiments were performed using serum from HF patients to treat HT-29 cells. Based on the known role of exosomes in serum miRNA transport (cited references), we tentatively hypothesize that exosomes may carry miR-221/222-3p in HF serum. However, this has not been verified in the present study. miR-221/222-3p may be present in HF serum exosomes, but future experiments (exosome isolation and miRNA detection) are needed to confirm this. Another limitation is the small sample size from a single-center cohort, the lack of data on HF severity indicators, and the absence of an external validation cohort, which may introduce selection bias. A further limitation is the restriction to HT-29 cells for validating the heart failure-cancer link, without exploration in other cancer models, which may limit insights into the differential roles of miR-221/222-3p in various cancers. It should be emphasized, however, that the study’s core aim was to test the specific role of heart failure-derived miR-221/222-3p as a key circulating factor, not to generalize about heart failure’s impact on all cancers.

## Conclusions

miR-221/222-3p has been identified as an important mediator of the progression of HF and colon cancer. miR-221/222-3p secreted by failing hearts promotes the proliferation of colon cancer cells. miR-221/222-3p could be potential targets for the treatment of colon cancer patients with heart failure.

## Data Availability

The datasets presented in this study can be found in online repositories. The names of the repository/repositories and accession number(s) can be found in the article/**Supplementary Material**.
