# Supplementary material for: miR-221/222-3p act as potential circulating factors in heart failure to stimulate cancer progression
Source: Front Oncol. 2026 Jan 12;15:1615422. doi: 10.3389/fonc.2025.1615422 (PMC12832461; doi:10.3389/fonc.2025.1615422)

Supplemental Figure 1. Mature sequences of miR-221/222-3p and their expression profiles in tissues. (A) Comparison between mature sequences of has-miR-221-3p and has-miR-222-3p, and the common seed sequences were enclosed in red frames. Gene CDKN1B was set as a typical example to illustrate base-complementation in two miRs. (B) The predicted secondary structures of pre-miR-221 and pre-miR-222 according to the sequences from miRbase.

A

Hsa-miR-221-3p: AGCUACAUUGUCUGCUGGGUUUC
Hsa-miR-222-3p: AGCUACAUCUGGCUACUGGGU

	Predicted consequential pairing of target region (top) and miRNA (bottom)	Site type	Context++ score	Context++ score percentile	Weighted context++ score	Conserved branch length	P_{CT}
Position 201-208 of CDKN1B 3' UTR<br>hsa-miR-221-3p	5' ...CUCUAAAAGCGUUGGAUGUAGCA...<br>3' CUUUGGGUCGUCUGUUACAUCGA	8mer	-0.52	99	-0.52	6.627	0.68
Position 201-208 of CDKN1B 3' UTR<br>hsa-miR-222-3p	5' ...CUCUAAAAGCGUUGGAUGUAGCA...<br>3' UGGGUCAUCGGUCUACAUCGA	8mer	-0.52	99	-0.52	6.627	0.68

B

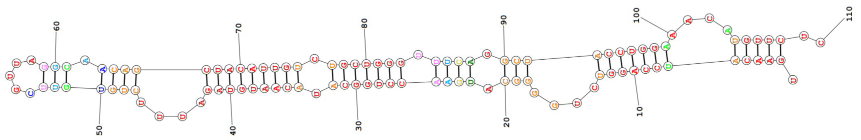

Pre-miR-221

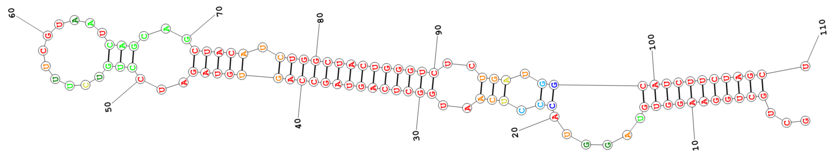

Pre-miR-222

Supplemental Figure 2. The human miRNA tissue atlas revealed a wide distribution of miR-221-3p among multiple tissues.

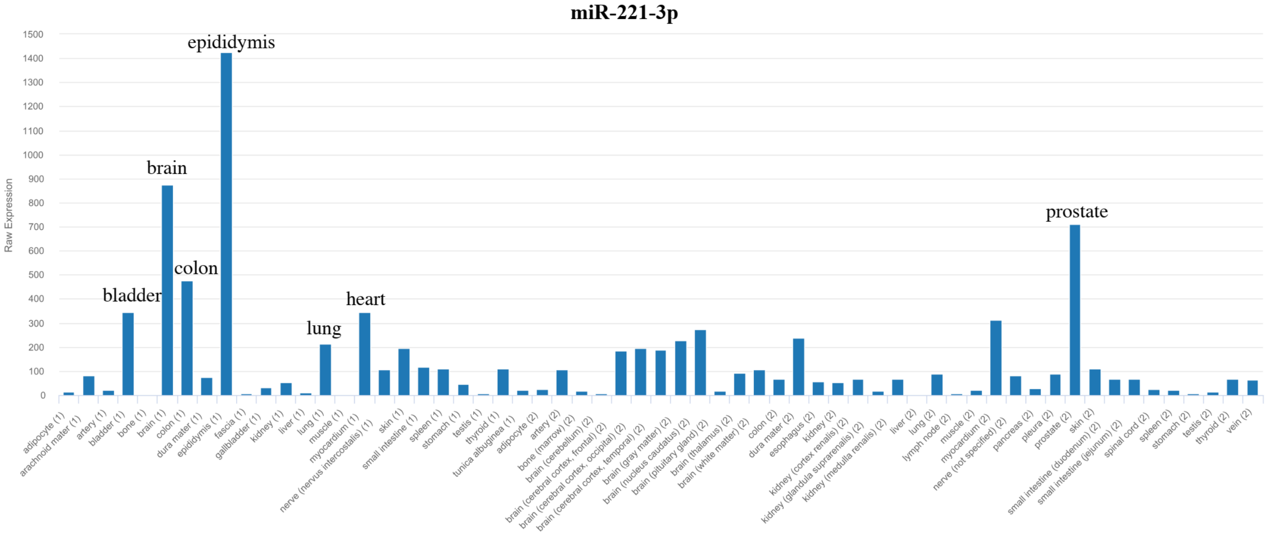

Supplemental Figure 3. The human miRNA tissue atlas revealed a wide distribution of miR-222-3p among multiple tissues.

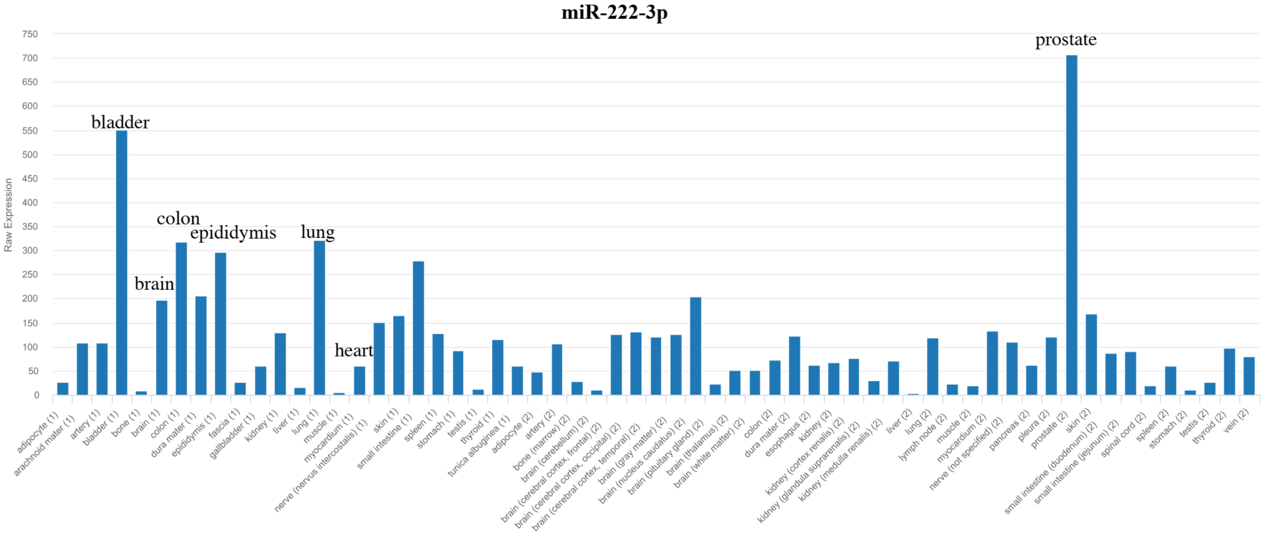

Supplemental Figure 4. PPI network of 43 target genes.

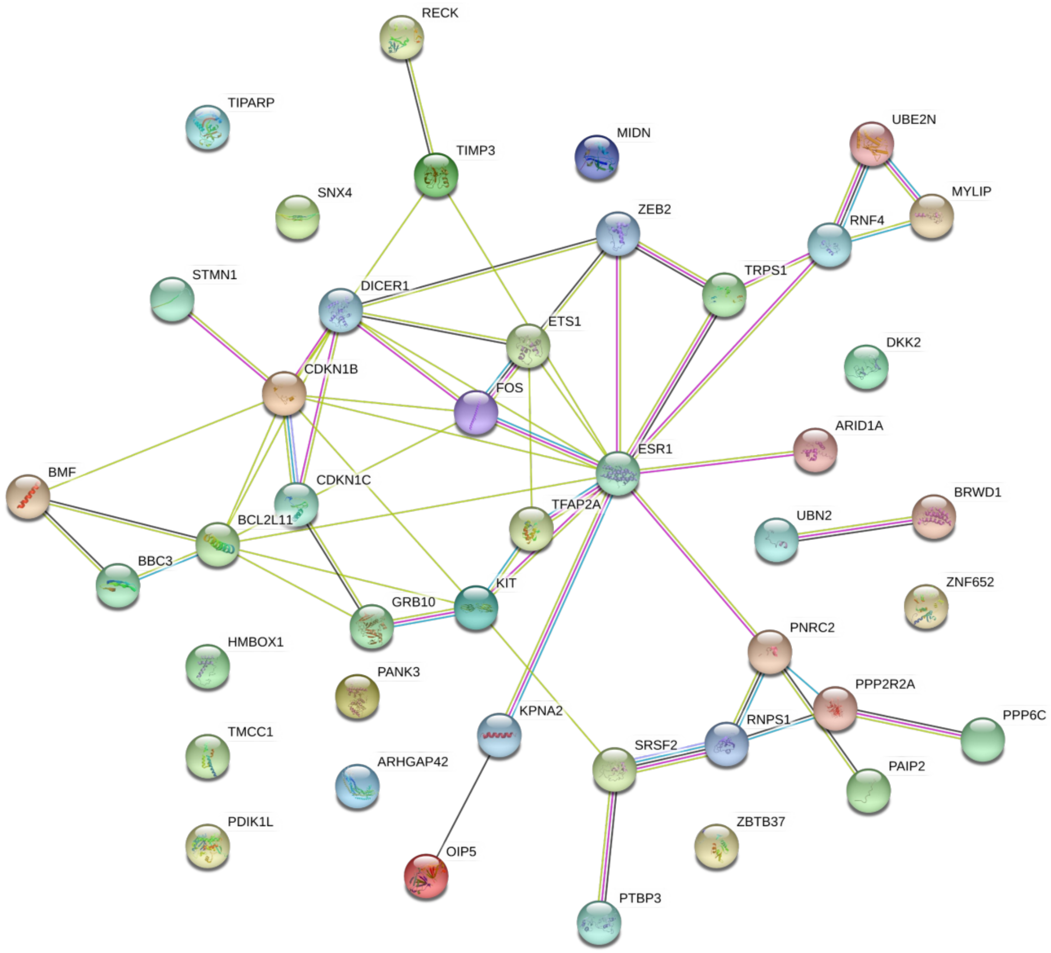

Supplemental Figure 5. PPI network combination of 15 and 43 disease-related genes of heart failure.

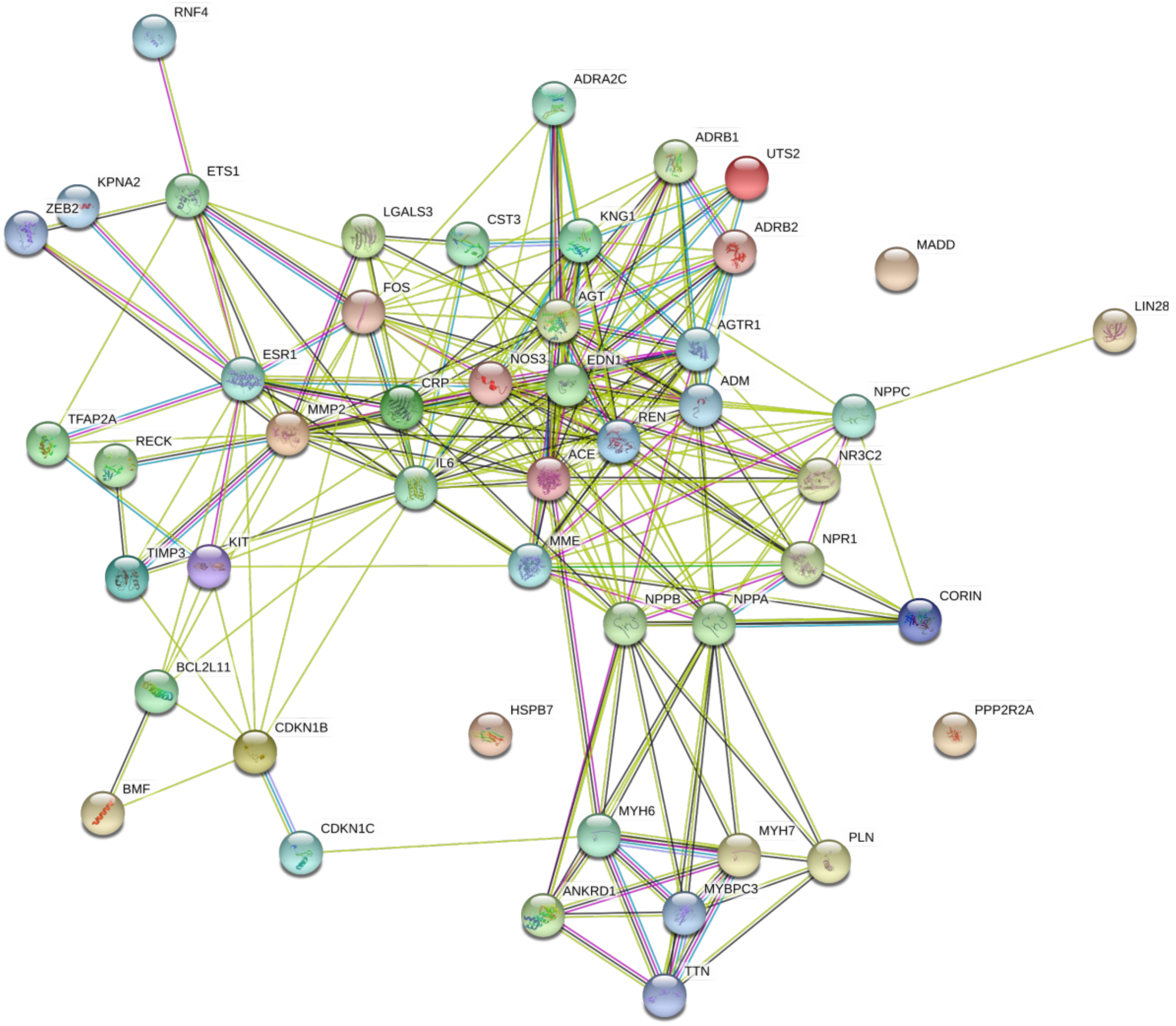

Supplemental Figure 6. PPI network of all the downregulated genes in GSE126092.

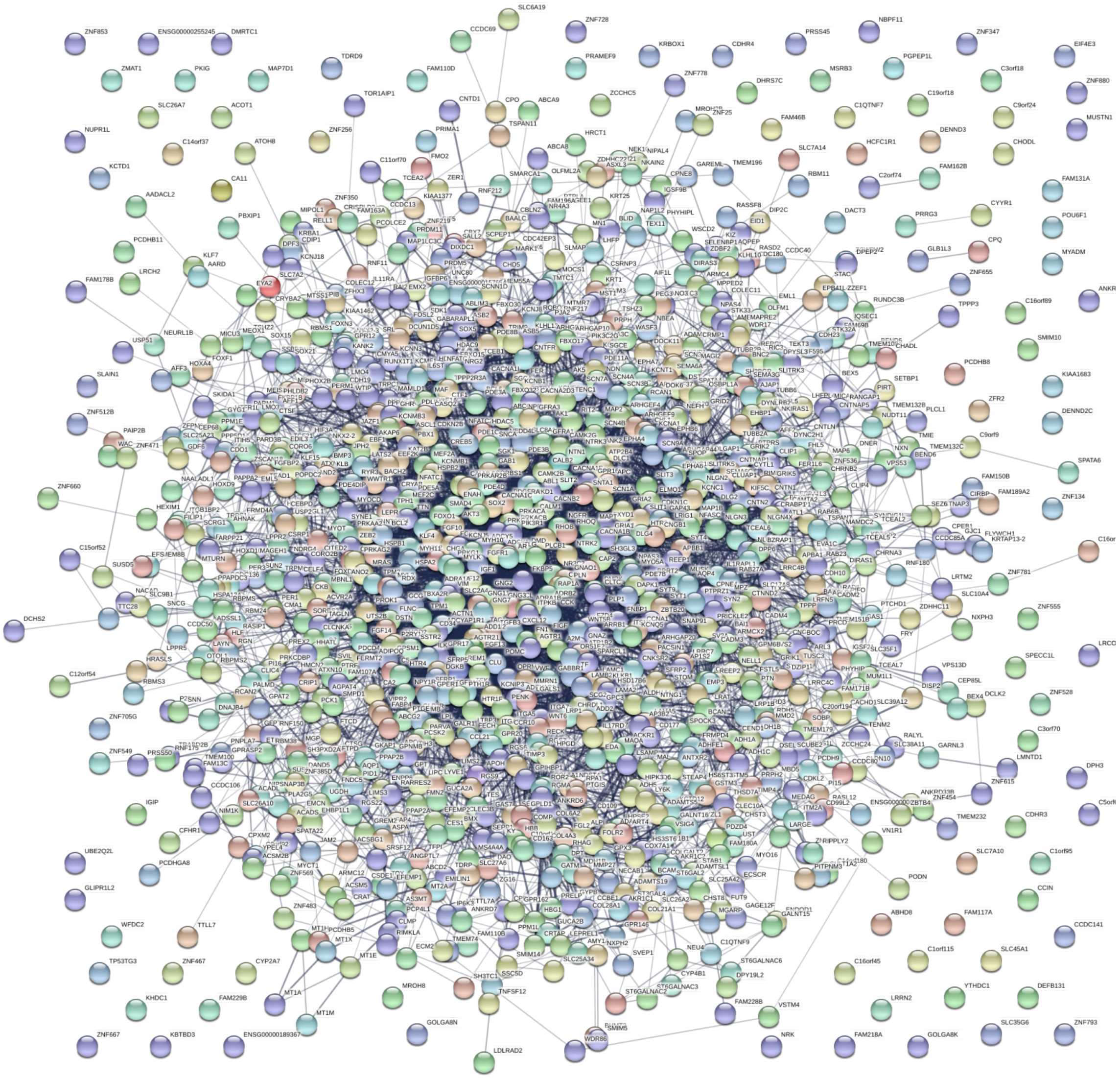

Supplement: Supplementary file 1 [file Image1.pdf]
