# Supplementary material for: miR-221/222-3p act as potential circulating factors in heart failure to stimulate cancer progression
Source: Front Oncol. 2026 Jan 12;15:1615422. doi: 10.3389/fonc.2025.1615422 (PMC12832461; doi:10.3389/fonc.2025.1615422)
Supplement: Supplementary file 4 [file DataSheet1.pdf]

| | Non-heart failure group (n=10) | Heart failure group (n=10) | P value |
|---|---|---|---|
| Gender (Male) | 6 (60%) | 8 (80%) | 0.628 |
| Age | 60.90±13.025 | 70.000±8.654 | 0.082 |
| BMI | 25.808±3.186 | 23.268±3.665 | 0.115 |
| SBP | 127.500±16.555 | 125.500±22.402 | 0.823 |
| WBC | 6.7740±0.918 | 6.244±1.857 | 0.429 |
| ALB | 38.530±2.108 | 36.580±3.680 | 0.163 |
| LVEF | 0.716±0.067 | 0.332±0.039 | <0.001 |
| NT-proBNP | 71.222±14.237 | 5631.000±6741.638 | 0.028 |
